# Global research on vitamin D and coronavirus disease 2019: A bibliometric and visualized study

**DOI:** 10.1097/MD.0000000000029768

**Published:** 2022-07-08

**Authors:** Muhammad Waseem Shah, Tauseef Ahmad, Muhammad Khan, Shafi Muhammad, Guiju Sun

**Affiliations:** a Key Laboratory of Environmental Medicine and Engineering of Ministry of Education, and Department of Nutrition and Food Hygiene, School of Public Health, Southeast University, Nanjing, China; b Department of Epidemiology and Health Statistics, School of Public Health, Southeast University, Nanjing, China; c Department of Biotechnology and Genetic Engineering, Centre for Human Genetics, Hazara University, Mansehra, Khyber Pakhtunkhwa, Pakistan; d Department of Biosciences, COMSATS University Islamabad, Tarlai Kalan, Islamabad, Pakistan.

**Keywords:** bibliometric analysis, COVID-19, Vitamin D, Web of Science

## Abstract

**Background and aim:**

Vitamin D play a substantial role in immune function, but little is known about its prevention in coronavirus disease 2019 (COVID-19). A detail bibliometric analysis of the published scientific literature indexed in Web of Science on vitamin D as a therapeutic option for the COVID-19 patients’ treatment is lacking. Thus, the current study was conducted to determine the key bibliometric indices and plot the global research on vitamin D and COVID-19.

**Methods:**

The Web of Science Core Collection database was utilized to retrieve publications on vitamin D and COVID-19. A Boolean search strategy was applied and the obtained data were exported to Microsoft Excel to generate relevant graphs. Furthermore, VOSviewer software version 1.6.17 for Windows was used to generate co-authorship countries, bibliographic coupling sources and co-occurrence keyword network visualization mapping. In addition, RStudio and Bibliometric online tool were used to generate WordCloud and thematic map, and intercountries relation map, respectively.

**Results:**

A total of 818 publications on vitamin D and COVID-19 were included in the final analysis. These publications were cited 10,713 times, with an H-index of 50. The number of publications and citations score from 2020 to November 2021 increased from 317 (2423 citations) to 501 (8290 citations). Delanghe JR and Speeckaert MM were the most prolific authors with 13 publications each. The most productive journal was *Nutrients* (n = 63). The most studied research area is nutrition dietetics. The most widely used author keywords were COVID-19 (n = 444), Vitamin D (n = 312), and SARS-CoV-2 (n = 190). The National Institute of Health and US Department of Health and Human Services were the leading funding agencies. Harvard University was the most active institution with 25 publications. The United States of America was the highly contributing and influential country in terms of publications (n = 203) and total link strength (n = 185).

**Conclusion:**

It was concluded that an increasing trend in the number of publications on vitamin D and COVID-19 has been observed. Significantly, the majority of the research has been conducted in developed countries. Most importantly, over the time, the direction of research has been changed and the recent trend topics are vitamin D deficiency, risk and infection, and vitamin D supplementation based on KeyWords Plus. The use of vitamin D supplement is one of the promising therapeutic options for COVID-19 treatment. Therefore, the current study not only highlight the global research trends but also provide standard bibliographic information for future studies.

## 1. Introduction

Around mid-December 2019, a cluster of pneumonia-like cases of unknown etiology and epidemiologically linked to a seafood market in Wuhan city, China, was reported. The global spread was attributed to human–human transmission, and afterward, the causative agent was shortly identified as a new strain of family *Coronaviridae* and *Orthocoronavirinae* subfamily.^[1,2]^ The newly identified virus was provisionally designated as 2019 novel coronavirus, renamed to the severe acute respiratory syndrome coronavirus 2 (SARS-CoV-2), and the disease caused known as coronavirus disease 2019 (COVID-19).^[3]^

Clinical manifestations due to COVID-19 range from mild infection to severe pneumonia. The critically ill patients have a higher risk of multiple organ complications and mortality despite receiving more interventions.^[4,5]^ The respiratory system is the primary target of COVID-19, (initially) the diagnostic tests included fever, dry cough, fatigue, and shortness of breath. Other reported systemic impacts and the complications of the COVID-19 infection includes cardiovascular, haematological, neurologic, rheumatological, and renal complications.^[6–11]^

The sprint to find possible new treatment for COVID-19 began as the outbreak posed the potential risk of turning into a pandemic. Initially, when declared a pandemic, the World Health Organization promoted basic hygiene rules (handwashing) and social distancing as the principle fighting tool to prevent the contraction of SARS-COV-2 infection.^[12]^ In addition, monoclonal antibodies, antisense RNA, and convalescent plasma transfusion have been evaluated as anti-COVID-19 treatment.^[13,14]^ Other various anti-COVID-19 strategies have also been suggested by different authors such as favipiravir, melatonin, various traditional Chinese medicines, montelukast, retinoids, and interferon-α2b.^[15–20]^

It is extremely difficult to compare the data on mortality due to COVID-19 across different countries. This disparity is due to multiple factors, for example, age, overall diet composition, socioeconomic status, quality of healthcare, etc. Another relevant key factor in this regard that received considerable attention during the COVID-19 pandemic is vitamin D.^[21]^ Findings from different studies have reported the influence of good vitamin D status, which shows less severe disease and mortality in COVID-19. Conversely, the lower serum levels of vitamin D might exacerbate the infection.^[22–26]^ It is suggested that individuals suffering from vitamin D deficiency have about 3 times higher chance of infection with SARS-CoV-2. Moreover, the probability of getting severe COVID-19 infection due to vitamin D deficiency is 5 times higher.^[27]^ Overall, the evidence regarding good vitamin D status and less severe COVID-19 is, however, not fully established yet. It is common to evaluate the progress of scientific community, that is, the researchers and their publications. This measurement has become a necessity and potentially impacts the funding decisions. The regular method for this purpose such as peer review is usually complemented with bibliometric methods.^[28]^ Bibliometric methods are a set of mathematical and statistical methods applied to analyze the quantity and quality of articles, books, and other forms of publications. With growing scientific discoveries and more and more research findings get publish, and are quoted by other researchers, bibliometric study become increasingly important.^[29]^ Bibliometric methods help to arrive in understanding the trends and communication patterns occurring in the literature relevant to a particular domain.^[30]^ Analysis of the “keywords search” has identified that articles relevant to multidisciplinary research have the highest impact.^[31]^

This present study is the first study of its kind to quantitatively analyze the key bibliometric indices and visual network mapping to provide an overview of the overall research trends, publication patterns, emerging research areas and worldwide collaborations in the field of vitamin D and COVID-19.

## 2. Methods

### 2.1. Study design

A retrospective bibliometric study was conducted.

### 2.2. Data source

Web of Science (WoS) is the world’s oldest and one of the largest abstract and citation databases.^[32]^ The WoS includes all abstract and indexing edition of chemistry, engineering, and medical and health sciences. In bibliometric types of studies, the WoS is one of the commonly used databases and easy to operate.^[33–35]^ A topic specific search was carried out on November 16, 2021, in the WoS Core Collection (WoSCC) database to retrieve the relevant information on COVID-19 and vitamin D. The search was limited to Science Citation Index Expanded (SCI-Expanded).

### 2.3. Search strategy and keywords

The Boolean search strategy was applied. The potential keywords used were “Coronavirus” OR “Novel coronavirus” OR “Coronavirus disease 2019” OR “COVID-19” OR “COVID” OR “Severe acute respiratory syndrome 2” OR “SARS-CoV-2” (Topic) and “Vitamin D” (Topic). The topic searches for title, abstract, author keywords, and keywords plus. The articles published between 2020 and 2021 (November) were included in the final analysis.

### 2.4. Data extraction and analysis

The data were downloaded in comma-separated values, Tab delimited and Plain text format. The basic data were extracted from the included documents were titles, authors name, languages, year of publications, journals, research areas, funding sources, top cited papers, institutions, and countries. The obtained data were exported into Microsoft Excel to generate relevant graphs. The values are presented in frequencies and percentages. Furthermore, the data were plotted for co-authorship countries, bibliographic coupling, and co-occurrence keywords network visualization mapping using VOSviewer software version 1.6.17 for windows (Leiden University, Leiden, The Netherlands). The VOSviewer is freely available software and is commonly used for network visualization mapping.^[36]^ The co-authorship countries network visualization illustrates the connection between co-authors and countries. The node of each country represents the contribution, whereas the link between the countries represents the collaboration among the authors from different countries. The larger the size of the keyword node, the greater the frequency of occurrence is. After plotting the data, different clusters were formed, and each color represented different clusters. Furthermore, WordCloud and thematic map were generated using RStudio biblioshiny package. In addition, intercountries collaboration map was generated using an online Bibliometric tool.

## 3. Results

### 3.1. Publication outputs

In this study, a total of 818 publications on vitamin D and COVID-19 were included in the final analysis. These publications were authored by 4077 authors (4.98 authors per publication) and published in 374 journals. The number of single-authored publications was 80. The total citation score of the analyzed publications (n = 818) was 10,713 (13.1 citations per paper). After excluding the self-citations, the total citation score was found to be 6221 (7.6 citations per paper).

### 3.2. Basic characteristics of top 10 most cited articles

The characteristics of top 10 most cited studies as of November 16, 2021 are presented in Table 1. A review article entitled as “Evidence that Vitamin D Supplementation Could Reduce Risk of Influenza and COVID-19 Infections and Deaths” and published in journal of *Nutrients* was ranked first with highest number of citations n = 524 (262 average per year citation), followed by an original article published in May 2020 and was cited 311 (155.5 average per year citation) times. The third ranked document was a review article which received total 233 citations with average per year citation of 116.5.

**Table 1.** Characteristics of top 10 most cited publications (as of November 16, 2021).

Rank	Total citations	Average per year	Document type	Journal	Title
1	524	262	Review	*Nutrients*	Evidence that vitamin d supplementation could reduce risk of influenza and COVID-19 infections and deaths
2	311	155.5	Article	*Aging Clinical and Experimental Research*	The role of vitamin D in the prevention of coronavirus disease 2019 infection and mortality
3	233	116.5	Review	*Nutrients*	Optimal nutritional status for a well-functioning immune system is an important factor to protect against viral infections
4	219	109.5	Editorial Material	*Clinical Nutrition*	ESPEN expert statements and practical guidance for nutritional management of individuals with SARS-CoV-2 infection
5	193	96.5	Article	*Journal of Steroid Biochemistry and Molecular Biology*	Effect of calcifediol treatment and best available therapy versus best available therapy on intensive care unit admission and mortality among patients hospitalized for COVID-19: a pilot randomized clinical study
6	184	92	Review	*Aging-US*	Why does COVID-19 disproportionately affect older people?
7	177	88.5	Article	*Nutrients*	25-Hydroxyvitamin D concentrations are lower in patients with positive PCR for SARS-CoV-2
8	161	80.5	Article	*JAMA Network Open*	Association of vitamin D status and other clinical characteristics with COVID-19 test results
9	158	79	Review	*Nutrients*	COVID-19: the inflammation link and the role of nutrition in potential mitigation
10	134	67	Review	*Nutrients*	Strengthening the immune system and reducing inflammation and oxidative stress through diet and nutrition: considerations during the COVID-19 crisis

### 3.3. Distribution of prolific authors, document types, publications language, and the leading journals

**Figure 1.**
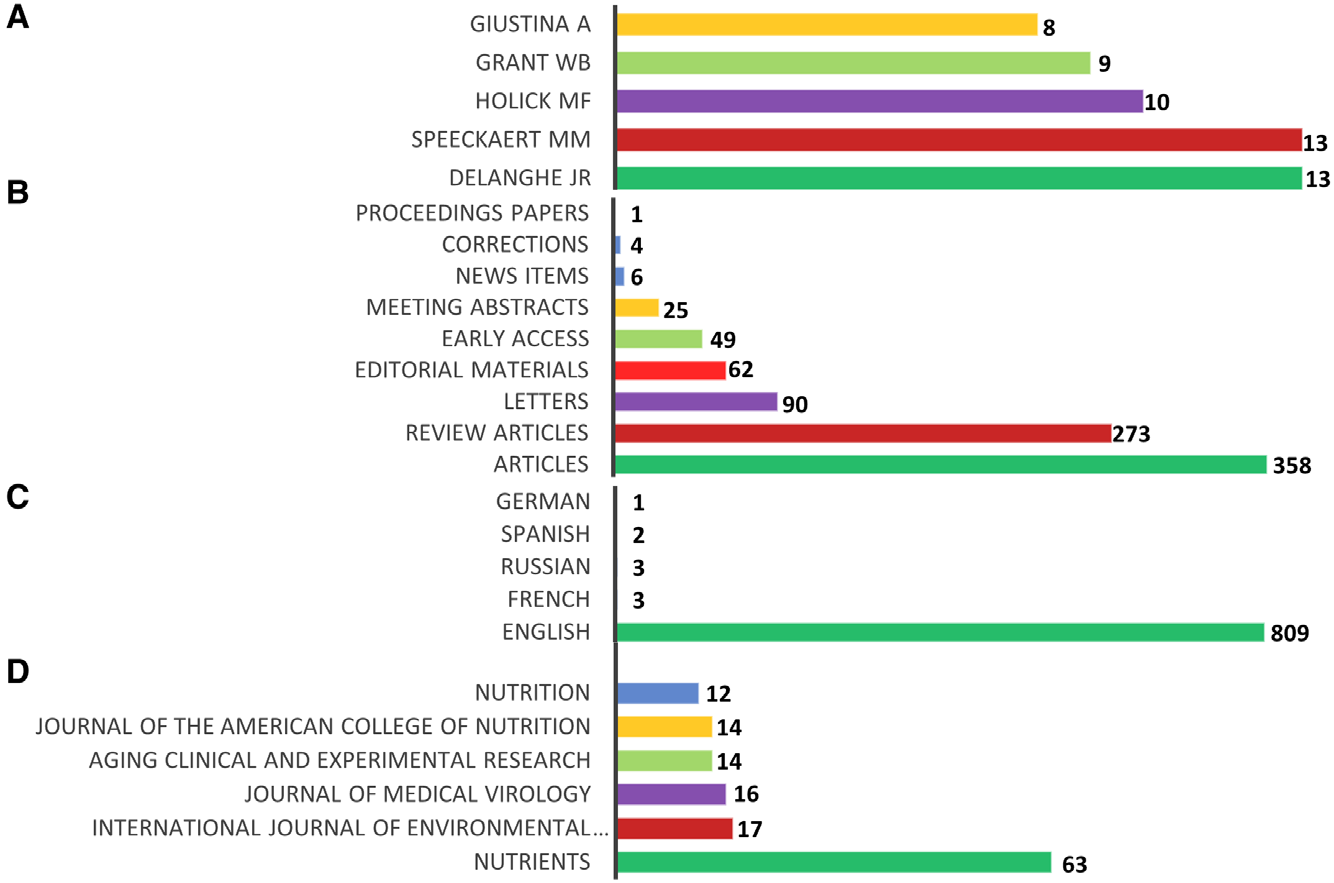
(A) Distribution of author’s publications, (B) document types, (C) publications language, and (D) leading journals in vitamin D and COVID-19 research. COVID-19 = coronavirus disease 2019.

The most prolific authors JRD and MMS were ranked first with (n = 13; 1.58%) publications each, followed by MFH (n = 10; 1.2%), WBG (n = 9; 1.10%), and AG (n = 8; 0.9%), as shown in Figure 1A. Most of the documents were original research articles (n = 358; 43.7%), followed by review articles (n = 273; 33.3%), and letters (n = 90; 11%), while the remaining document types (Editorial materials, Early access, Meeting abstracts, News items, Corrections, and Proceeding papers) were found to be <10% (Fig. 1B). Most of the documents were published in English language (n = 98%), as shown in Figure 1C. The most productive journals were *Nutrients* with 63 (7.7%) publications and *International Journal of Environmental Research and Public Health* (n=17; 2%), as shown in Figure 1D.

### 3.4. Most studied research areas, leading funding agencies, country of origin, and institutions

**Figure 2.**
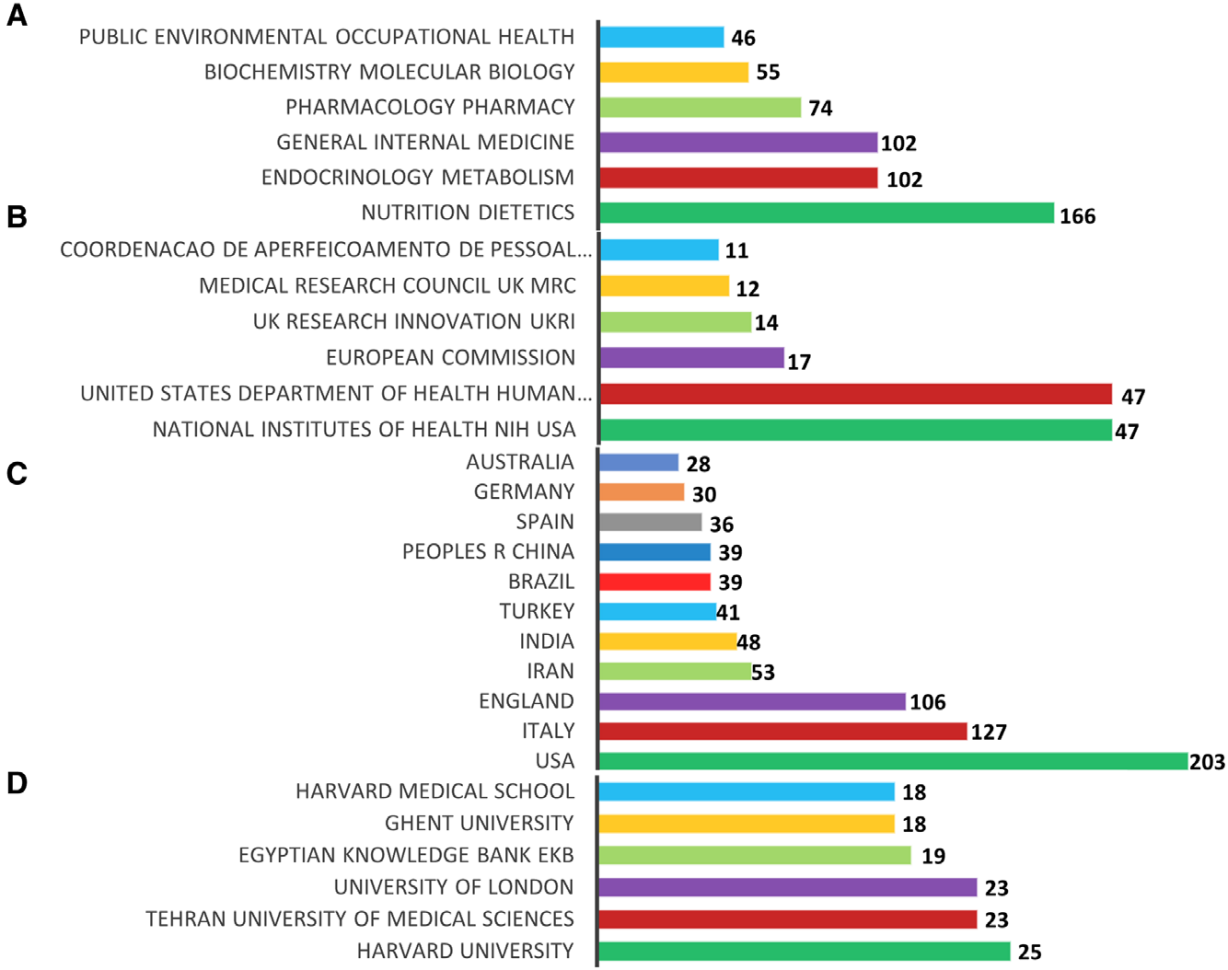
(A) Most studied research areas, (B) leading funding agencies, (C) country of origin, and (D) institutions in vitamin D and COVID-19 research. COVID-19 = coronavirus disease 2019.

Details of the most studied research areas, funding agencies, top 10 countries of origin, and the top 5 most productive institutions are presented in Figure 2A–D. The most studied research area was nutrition dietetics with the record count of 166 (20.29%), followed by endocrinology metabolism and general internal medicine (n = 102; 12.49%), and pharmacology pharmacy (n = 74; 9.04%). The National Institute of Health, United States of America (USA), and the US Department of Health and Human Services were the leading funding agencies. The United States was the most productive country produced 203 (24.8%) publications, followed by Italy (n = 127; 15.52%), England (n = 106; 12.95%), and Iran (n = 53; 6.47%). Harvard University was ranked first with n = 25 (3.05%) publications, followed by Tehran University of Medical Sciences, and University of London with an equal number of contributions (n = 23; 2.18%).

### 3.5. Co-authorship countries network visualization mapping

**Figure 3.**
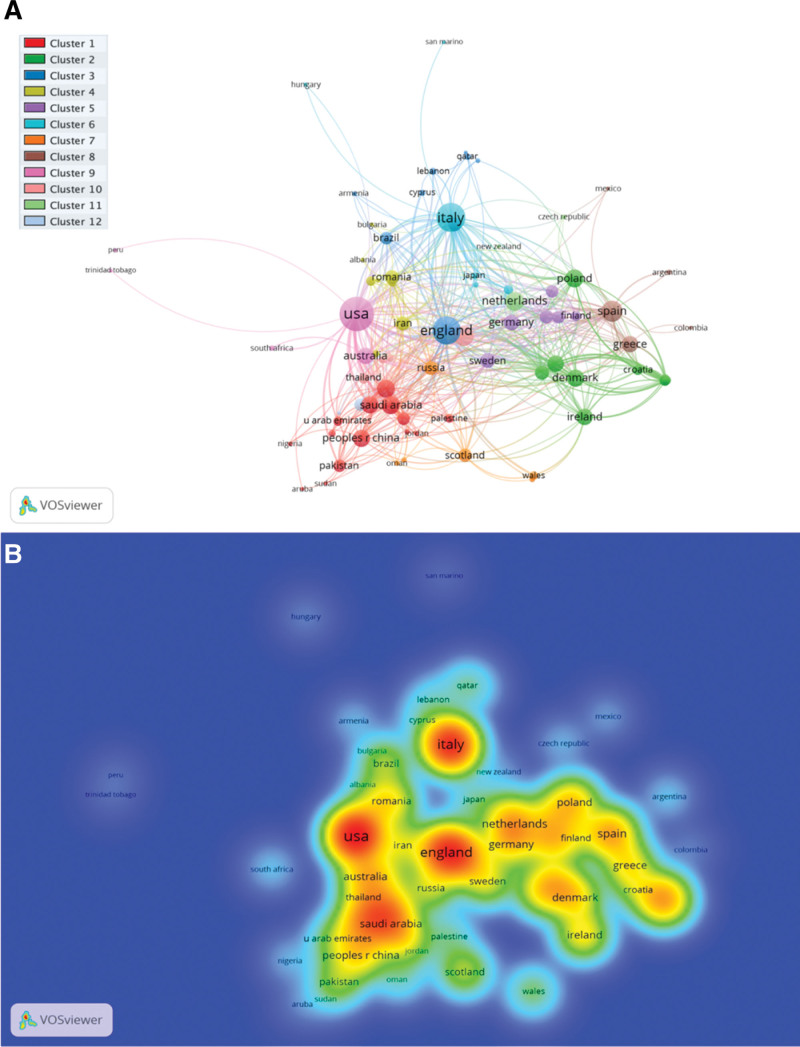
Co-authorship countries network visualization (A); density visualization (B) based on total link strength.

A total of 80 countries were plotted for co-authorship countries network visualization mapping as shown in Figure 3A. A total of 12 clusters were formed, and each color designates different cluster. The cluster 1 consists of 14 items, followed by cluster 2 (12 items), cluster 3 (9 items), cluster 4 (8 items), cluster 5 (6 items), cluster 6 (6 items), cluster 7 (6 items), cluster 8 (5 items), cluster 9 (5 items), cluster 10 (4 items), cluster 11 (3 items), and cluster 12 (2 items). The United States had the highest total link strength (185), followed by Italy (133), England (126), Spain (73), and France (67). Furthermore, the co-authorship countries' density visualization mapping is presented in Figure 3B.

### 3.6. Intercountries collaboration mapping

**Figure 4.**
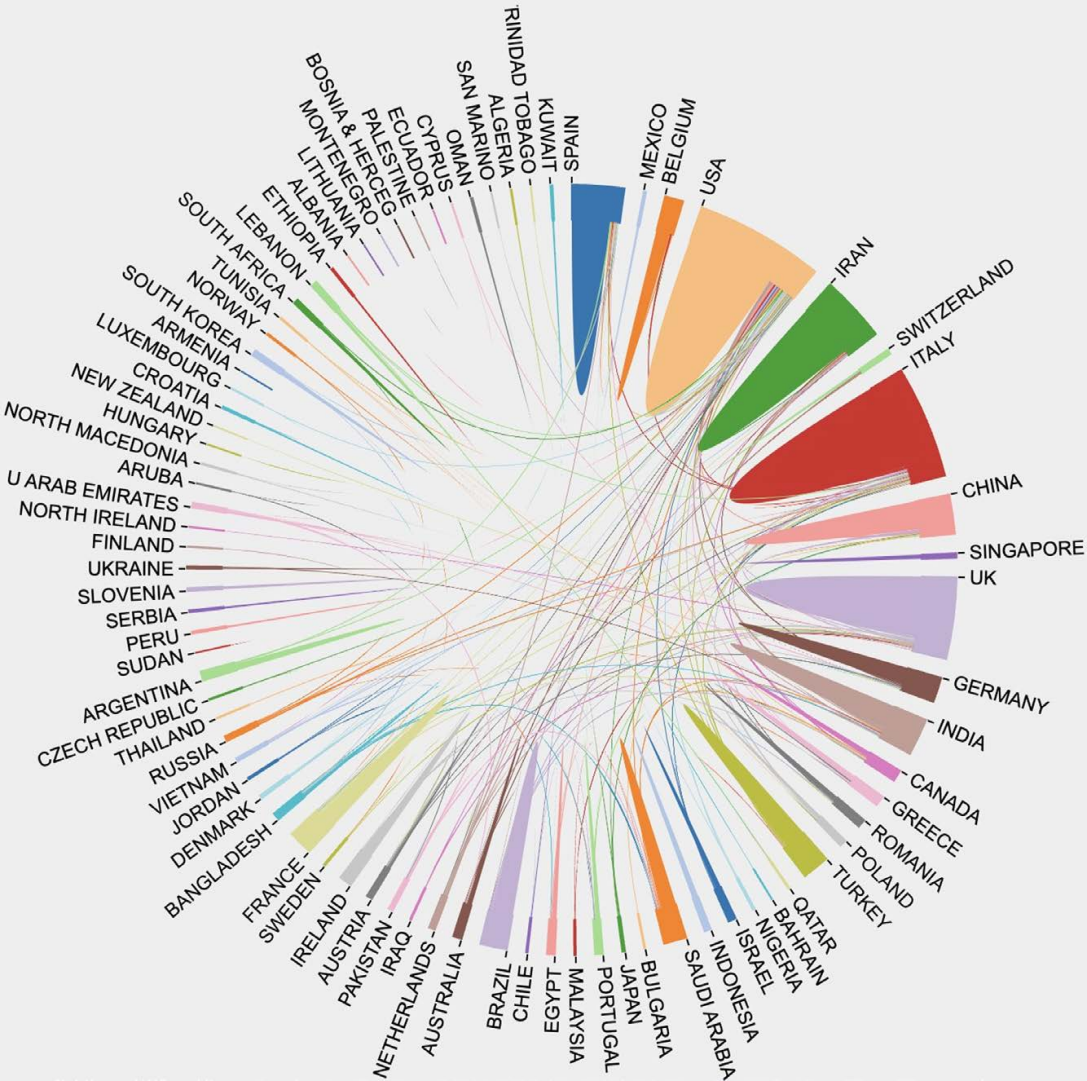
Intercountries collaboration mapping.

The intercountries or regions collaboration is presented in Figure 4, which indicated that among all the countries involved in vitamin D and COVID-19 research shows that the United States was found to be the highly contributing and collaborative country followed by Italy and the United Kingdom.

### 3.7. Co-occurrence keywords network visualization mapping

**Figure 5.**
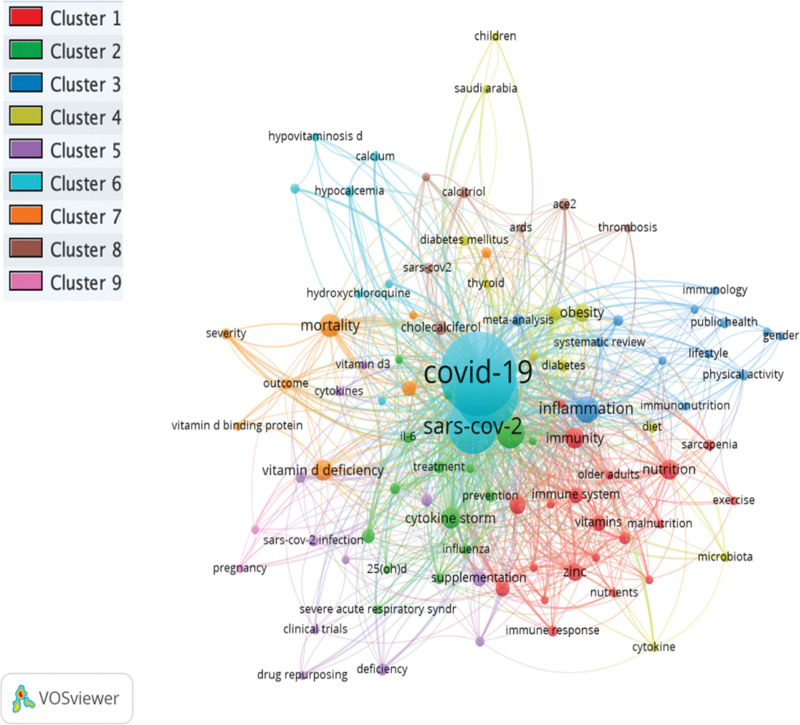
Co-occurrence author keywords network visualization based on the number of occurrences.

**Figure 6.**
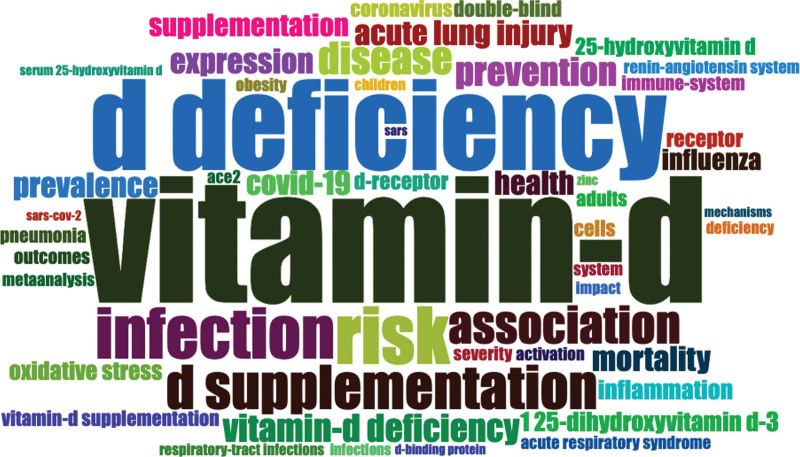
WordCloud map of KeyWord Plus.

The minimum number of occurrences of a keyword was fixed at 5. Of the total author keywords 1398, only 97 met the threshold and were plotted. The most widely used author keywords were COVID-19 (n = 444), vitamin D (n = 312), SARS-CoV-2 (n = 190), coronavirus (n = 63), and inflammation (n = 45) as shown in Figure 5. A total of 9 clusters were formed and each color represents different cluster. The cluster 1 consists of 20 items, followed by cluster 2 (15 items), cluster 3 (13 items), cluster 4 (11 items), cluster 5 (11 items), cluster 6 (10 items), cluster 7 (8 items), cluster 8 (6 items), and cluster 9 (2 items). In addition, the KeyWord Plus WordCloud map is presented in Figure 6. The most appeared keywords were vitamin D (n = 163), D deficiency (n = 117), risk (n = 74), infection (n = 66), D supplementation (n = 57), and association (n = 49).

### 3.8. Bibliographic coupling sources

**Figure 7.**
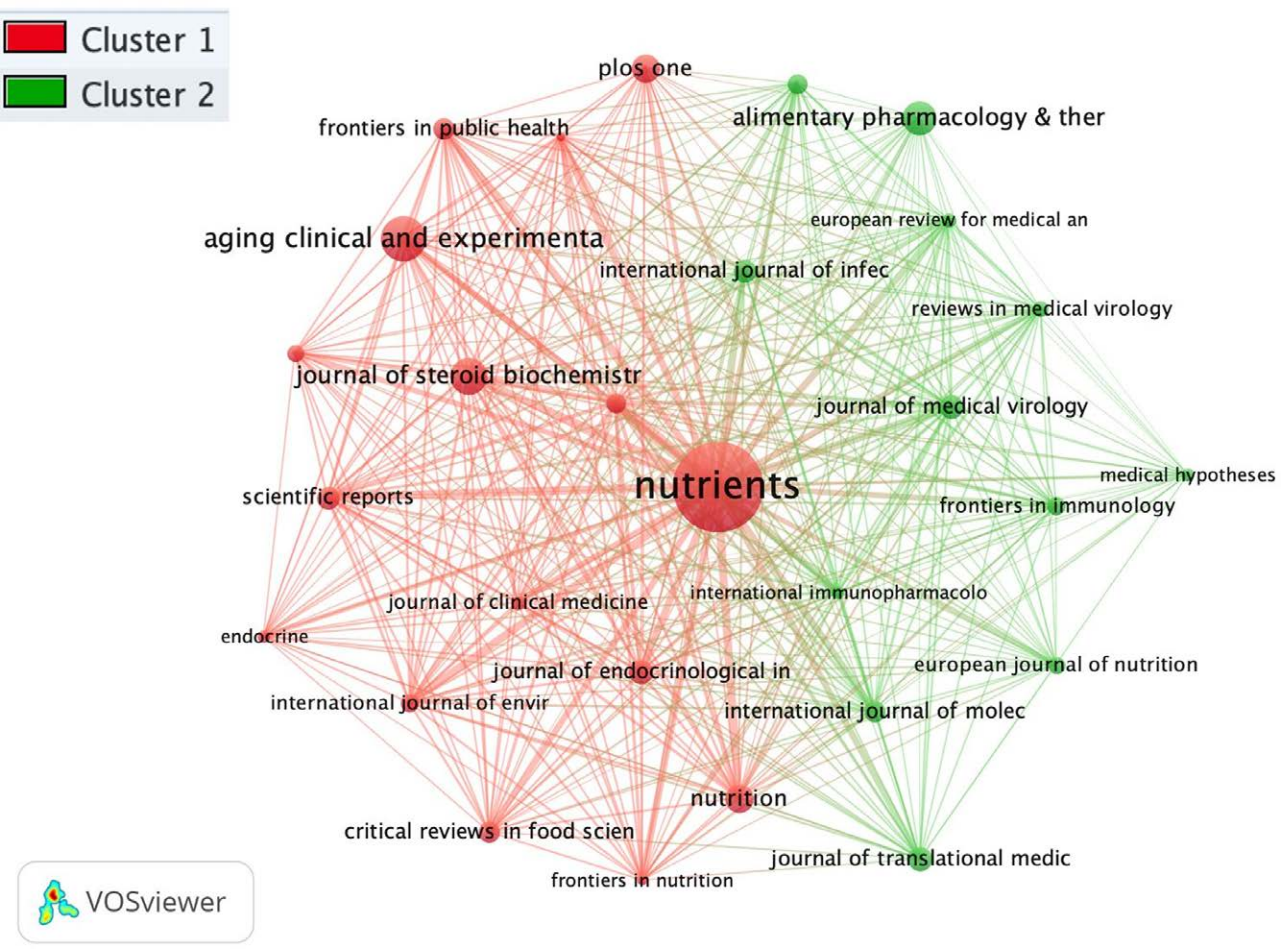
Bibliographic coupling sources network visualization.

The obtained data were plotted for bibliographic coupling sources based on citations as shown in Figure 7. The minimum number of documents of a source was selected at 5. The network visualization analysis shows that *Nutrients* was the leading journal based on the number of documents, citations, and total link strength. A total of 2 clusters were formed; the cluster 1 consists of 16 items and cluster 2 consists of 12 items.

### 3.9. Thematic map of published literature

**Figure 8.**
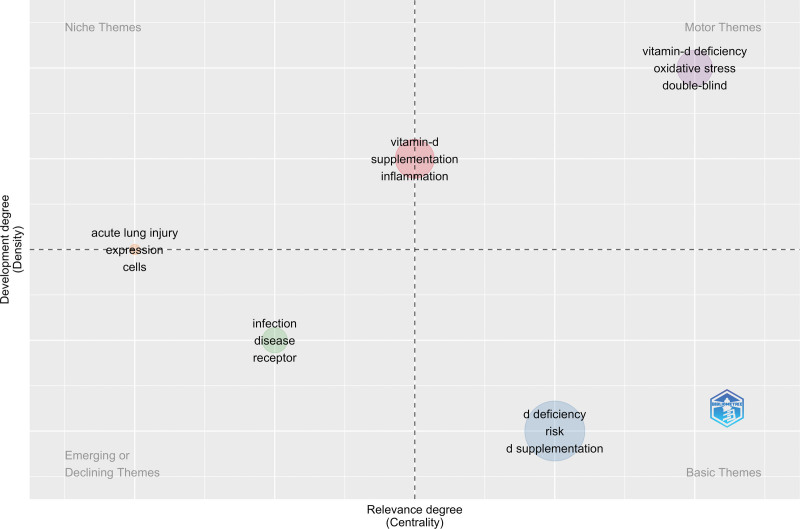
Thematic map of the published literature on COVID-19 and vitamin D based on KeyWords Plus. COVID-19 = coronavirus disease 2019.

The thematic map of published literature on COVID-19 and vitamin D is presented in Figure 8. The data were plotted into 4 themes/quadrants: Motor Themes (bridge between other topics), Niche Themes (highly developed topics), the Basic Themes (basic and transversal topics currently under development), and Emerging or Declining Themes (emerging topics). The centrality represents the power/strength of association between keywords in one cluster with another, while the density shows that aggregate power/strength of the association between the keywords in the same cluster.^[37]^ The basic research focused on vitamin D deficiency, risk, supplementation, and prevention of COVID-19. The research direction is shifted from the basic to other topics including vitamin D, supplementation, inflammation, oxidative stress, obesity, impact and mechanism, acute lung injury, and expression.

## 4. Discussion

Previous studies show that low levels of vitamin D have been associated with an increased risk of viral upper respiratory tract infections and pneumonia.^[38]^ The severity of disease in COVID-19 patients is determined by acute respiratory distress syndrome, microvascular thrombosis, and myocarditis, which involve underlying inflammation.^[39]^

The deficiency of vitamin D disturbs the functions of immune system because it exerts an immunomodulation role and enhances host immune responses by antiviral peptides secretions.^[40–42]^ Some of the recent studies hypothesized that the deficiency of vitamin D may increase the risk of COVID-19 severity and mortality as its deficiency may compromise the immune functions of respiratory system.^[23,43]^ However, some of the studies have also determined the correlation between vitamin D levels and the severity and mortality of COVID-19.^[44–49]^ Trial reports have revealed that vitamin D supplementation in the COVID-19 infection possess a potential impact by reducing the risk of COVID-19. Considering the fact that COVID-19 outbreak occurred in winter, the serum vitamin D levels are usually at its lowest, and its deficiency leads to acute respiratory distress syndrome and higher mortality rates with both age and chronic disease comorbidity.^[23]^ Moreover, vitamin D supplementation for patients at high risk of respiratory tract infection in a randomized control trial has shown reduction in both the symptoms and need for antibiotic therapy.^[50]^

To the best of our knowledge, this is the first bibliometric analysis to explore the global research on the association of vitamin D with COVID-19, indexed in the WoSCC database. Bibliometric studies play a significant role in providing the referral point for the researchers, policymakers, and medical practitioners.^[51]^ The study documented the most dynamic authors and countries, most frequent subject areas, most productive authors, and journals and citation patterns. There is an increasing number of publications in 2021 (as of November 16, 2021) owing to raise efforts for the treatment of COVID-19 which led to enhance research trend in the field.

In present study, the most highly cited article was identified as a review article published in April 2020, which documented the role of vitamin D supplementations in reducing the risk of COVID-19 and respiratory tract infections and influenza.^[23]^ The second most highly cited study that attracted more attention was an original article published in May 2020. This study primarily assessed the association between mean levels of vitamin D in several countries and the mortality triggered by COVID-19, while the possibility of association between the mean vitamin D levels in several countries and the number of COVID-19 cases was also identified.^[52]^

The United States was the leading contributor in publications on the topic of our search strategy followed by Italy, the United Kingdom, and Iran. This study has similar trends with many other bibliometric studies of diverse disciplines that confirmed the United States as a global leader both quantitative and qualitatively.^[53–56]^ This dominance can also be because of the extremely devoted researchers and the availability of huge research funds and well-established research laboratories.^[57]^ In some cases, the number of articles published by any country does not represent the highest number of citations. This unveils the fact that every article will not necessarily get the good citations rather than it depends on the content, usefulness or applications, originality, and any new aspect in the field. Top 3 most prolific authors have published a significant number of publications. It has inserted a directly proportional effect on citations. Higher the number of publications got more citations.

Harvard University was the institution that published the highest number of articles related to vitamin D and COVID-19 followed by Tehran University of Medical Sciences and University of London. In current study, low- and middle-income countries are among top 5 and top 10. This trend shows the fact that the scientist from low- and middle-income countries are proactive as a high number of publications in current study are the review articles which does not need any laboratory facility or finance. This has led the researchers from low- and middle-income countries to contribute more. This also represents that the nonavailability of budget affects the capabilities of the scientist with limited or no resources. To combat this, the researchers in developing countries should collaborate with researchers from developed countries to learn new techniques and share the abilities and resources.

The role of funding agencies and research organizations in the promoting science and research is of key importance.^[58]^ The results show that most of the funding agencies were from the United States and other developed countries. The finding of our study is in line with other studies.^[59]^ Journals are considered as an important tool for the dissemination of research. The quality and prestige of a journal play major role in transmitting the research to the concern segment of the society.^[56]^ Most of the articles have been published in *Nutrients* (impact factor [IF]: 5.42), *Aging Clinical and Experimental Research* (IF: 3.63), and *Clinical Nutrition* (IF: 7.32). This trend represents that generally the authors prefer the relevancy over the IF of a journal. Moreover, specialty of a journal also plays important role in attracting the relevant studies from different regions of the world. The access of reader to the article also affects the number of citations. Due to the limited budgets, certain universities usually have a limited subscription. As a result, scientific community can only reach to the open-access articles. In current study most of the top 10 journals are open access.

Our findings highlight the increased research activity on one of the most concern health issues with respect to current pandemic, considered growing importance in several countries and need further studies for the confirmation of laboratory-based and clinical results of the studies. A large sample size should be taken, and phase III of clinical trial must be carried out before drawing any conclusion.

## 5. Study limitations

The present study used WoSCC database which is a reliable source of international peer-reviewed journals^[60]^ for bibliometric research and citation analysis. Despite its contributions, the current study has certain limitations. First, a single database was used which may bias the citations count and publications frequency by using other databases such as Google Scholar, PubMed, and Scopus. Second, the self-citations influence was not excluded.

## 6. Conclusion

This is the first bibliometric and visualized study to analyze the global research trends and development in vitamin D as a therapeutic option for COVID-19 patients’ treatment. It was concluded that an increasing trend in the number of publications on vitamin D and COVID-19 has been observed. Significantly, the majority of the research has been conducted in developed countries. Most importantly, over the time, the direction of research has been changed and the recent trend topics are vitamin D deficiency, risk and infection, and vitamin D supplementation based on KeyWords Plus. The use of vitamin D supplement is one of the promising therapeutic options for the COVID-19 treatment. Therefore, the current study not only highlight the global research trends but also provide standard bibliographic information for future studies.
